# α7 nicotinic acetylcholine receptor signaling modulates the inflammatory phenotype of fetal brain microglia: first evidence of interference by iron homeostasis

**DOI:** 10.1038/s41598-017-09439-z

**Published:** 2017-09-06

**Authors:** M. Cortes, M. Cao, H. L. Liu, C. S. Moore, L. D. Durosier, P. Burns, G. Fecteau, A. Desrochers, L. B. Barreiro, J. P. Antel, M. G. Frasch

**Affiliations:** 1 Department of Obstetrics and Gynaecology and Department of Neurosciences, CHU Ste-Justine Research Centre, Faculty of Medicine, Montreal, Canada; 2 Animal Reproduction Research Centre (CRRA), Faculty of Veterinary Medicine, Université de Montréal, Montréal, QC Canada; 3 Neuroimmunology Unit, Montréal Neurological Institute, McGill University, Montréal, QC Canada; 4 Department of Clinical Sciences, Faculty of Veterinary Medicine, Université de Montréal, St-Hyacinthe, QC Canada; 5 Department of Pediatrics, CHU Ste-Justine Research Centre, Faculty of Medicine, Université de Montréal, Montréal, QC Canada; 6 Department of Obstetrics and Gynecology, University of Washington, Seattle, WA USA

## Abstract

Neuroinflammation *in utero* may result in life-long neurological disabilities. Microglia play a pivotal role, but the mechanisms are poorly understood. No early postnatal treatment strategies exist to enhance neuroprotective potential of microglia. We hypothesized that agonism on α7 nicotinic acetylcholine receptor (α7nAChR) in fetal microglia will augment their neuroprotective transcriptome profile, while the antagonistic stimulation of α7nAChR will achieve the opposite. Using an *in vivo* - *in vitro* model of developmental programming of neuroinflammation induced by lipopolysaccharide (LPS), we validated this hypothesis in primary fetal sheep microglia cultures re-exposed to LPS in presence of a selective α7nAChR agonist or antagonist. Our RNAseq and protein level findings show that a pro-inflammatory microglial phenotype acquired *in vitro* by LPS stimulation is reversed with α7nAChR agonistic stimulation. Conversely, antagonistic α7nAChR stimulation potentiates the pro-inflammatory microglial phenotype. Surprisingly, under conditions of LPS double-hit an interference of a postulated α7nAChR - ferroportin signaling pathway may impede this mechanism. These results suggest a therapeutic potential of α7nAChR agonists in early re-programming of microglia in neonates exposed to *in utero* inflammation via an endogenous cerebral cholinergic anti-inflammatory pathway. Future studies will assess the role of interactions between inflammation-triggered microglial iron sequestering and α7nAChR signaling in neurodevelopment.

## Introduction

Brain injury acquired antenatally remains a major cause of long-term neurodevelopmental sequelae in children and adults^1^. Although the etiology of antenatal brain injury is undoubtedly multifactorial, there is growing evidence for a role of maternal and fetal infection and inflammation^2–4^, which is supported by animal studies^2, 5, 6^. Both systemic and neuroinflammation have been implied as important pathophysiological mechanisms acting independently to cause fetal brain injury or contributing to *in utero* asphyxial brain injury with consequences for postnatal health^7, 8^.

The main cause of fetal inflammation, chorioamnionitis, is a frequent (10% of all pregnancies, up to 40% of preterm births) and often subclinical fetal inflammation associated with ~9fold increased risk for cerebral palsy spectrum disorders with life-lasting neurological deficits and an increased risk for acute or life-long morbidity and mortality, inversely correlated with gestational age at delivery^8–10^.

In addition to short-term brain damage, neuroimmune responses to *in utero* infection may also have long-term health consequences. In adults, exposure to inflammatory stimuli can activate microglia (glial priming^11, 12^). Confronted with a renewed inflammatory stimulus, they can sustain chronic or exaggerated production of pro-inflammatory cytokines associated with postnatal neuroinflammatory diseases such as Multiple Sclerosis or sustained cognitive dysfunction (“second hit” hypothesis)^12–14^.

α7 nicotinic acetylcholine receptor (α7nAChR) signaling in microglia may be involved in modulating TNF-α release to push microglia towards a neuroprotective role under conditions of lipopolysaccharide (LPS) exposure^15–17^.

We hypothesized that agonistic stimulation of α7nAChR in fetal microglia will augment their neuroprotective profile, while the antagonistic stimulation of α7nAChR will achieve the opposite. Using a novel *in vivo* - *in vitro* model of developmental programming of neuroinflammation induced by LPS, we validate this hypothesis in primary fetal sheep microglia cultures exposed to LPS in presence of a selective α7nAChR agonist or antagonist.

Iron homeostasis is tightly intertwined with control of inflammation^18^. Iron deficiency is the most common form of nutrient deficiency worldwide. According to the World Health Organization, it affects nearly 2 billion people and up to 50% of women who are pregnant^19^. At birth, 25–85% of premature babies are iron deficient and all will become iron deficient after birth, if not adequately supplemented, especially in developing countries^20^. Iron is essential for neonatal and long-term cognitive and physical development^21, 22^.

We hypothesized that fetal or early postnatal inflammation may result in hepcidin-mediated intracellular microglial iron sequestration which polarizes microglia toward an inflammatory phenotype. Our present findings suggest a novel signaling system involving the α7nAChR and the hepcidin-ferroportin signaling cascades.

## Results

### Primary fetal sheep microglia culture

**Figure 1:**
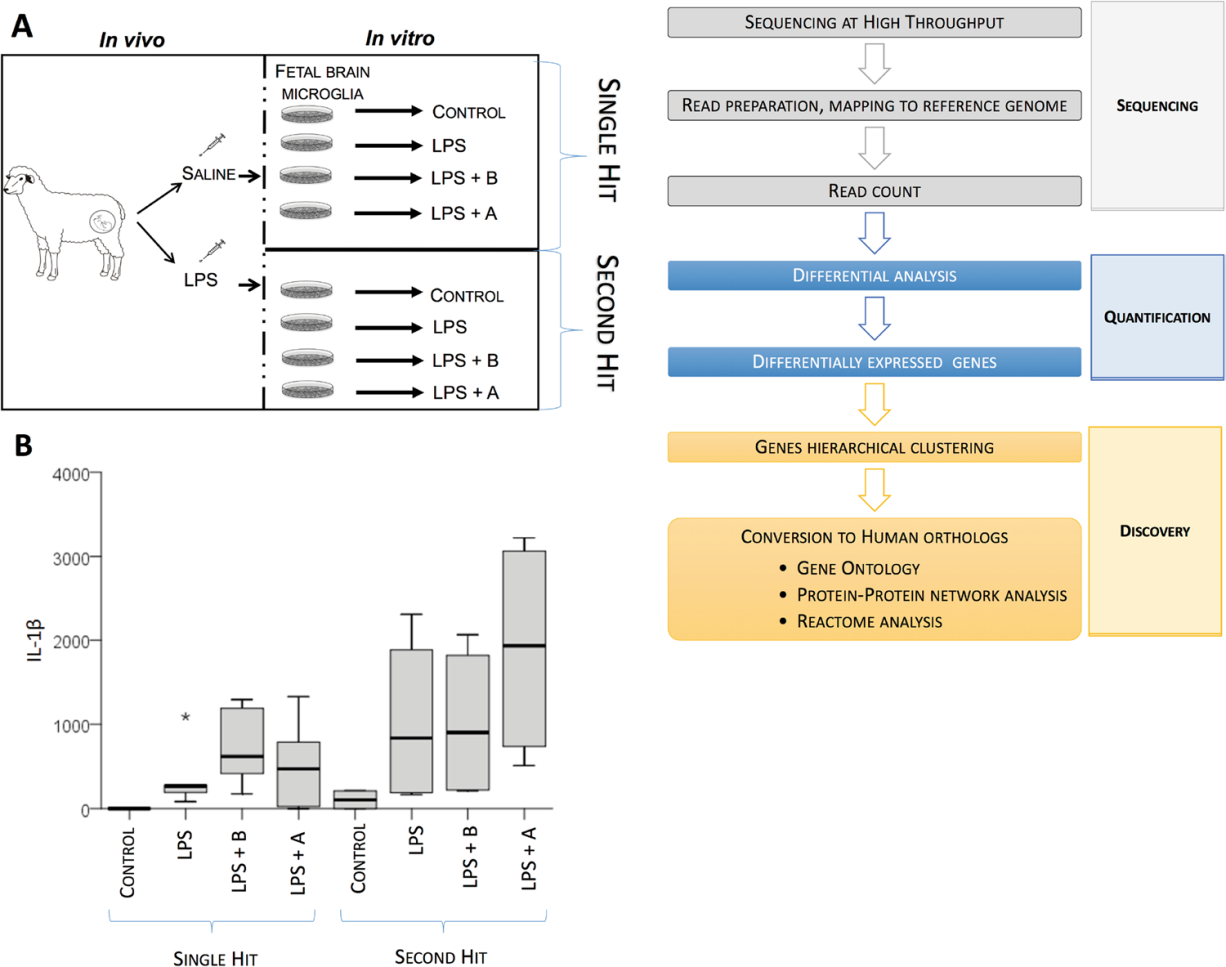
Experimental design of modulating α7nAChR signaling in a double-hit fetal sheep model. (**A**) *In vivo, in vitro* and RNAseq experiments are illustrated. *In vivo* study includes Control (saline) or LPS-exposed sheep fetuses. For the *in vitro* study, cultured cells were derived from an *in vivo* Control animal, named as Naïve or from an *in vivo* LPS-exposed animal, named as second hit (SH). There are 8 experimental groups: naïve Control (NC), naïve LPS (NL), naïve exposed to α-Bungarotoxin (NB), naïve exposed to AR-R17779 (NA), and each respective second-hit groups (SH). For RNAseq data comparisons, the group SH + agonist (SHA) was excluded. (**B**) Supernatant cytokine IL-1β response. * represents an outlier outside 95^th^ percentile. GEE model results are presented in text and no significance marks are provided in the figure. Briefly, we found significant main and interaction term effects (p < 0.05) for LPS and drug treatment and the contribution of *in vivo* LPS exposure, *i.e*., SH effect on IL-1β secretion profile.

*In vitro* studies were conducted in primary cultures derived from six controls (naïve control, NC) and from two *in vivo* LPS-exposed animals (second hit, SH) in 1-2 *in vitro* replicates from each animal depending on cell numbers obtained (Fig. 1A). First, we investigated cytokine secretion properties of microglial cultures in the absence or presence of LPS. Methodology and results are presented elsewhere^23^. Second, we studied IL-1β secretion profile (Fig. 1B) in response to LPS accompanied by co-incubation with α7nAChR agonist AR-R17779 (naïve agonist, NA or SH agonist, SHA) and the α7nAChR antagonist α-Bungarotoxin (NB or SHB).

The cytokine IL-1β secretion profile showed a non-random distribution pattern (p < 0.001, main term “group”), but not for the main term “hits” (p = 0.122). The latter main term “hits” identified each group as having or not having been exposed to an *in vivo* hit, *i.e*., testing for the SH effect on IL-1β secretion profile without accounting for the experimental group. LPS exposure led to IL-1β rise (p = 0.020) which was non-randomly changed by α7nAChR agonism (p < 0.001), but not by antagonism (p = 0.801).

The generalized estimating equations (GEE) model exploring the contribution of the second hit to the IL-1β secretion profile revealed a significant interaction between the four experimental groups (control, LPS, LPS with antagonist and agonist pre-treatments) and the presence or absence of two hits (p < 0.001 for interaction term “group” * “hits”). Specifically, without the preceding *in vivo* hit, α7nAChR agonism reduced the effect of this heightened IL-1β secretion (p = 0.028). Surprisingly, with the preceding *in vivo* hit, *i.e*., in the SH groups, α7nAChR agonism amplified the effect (p = 0.028). That is, *in vivo* exposure to LPS reversed the effect of the agonistic α7nAChR stimulation. Meanwhile, for α7nAChR antagonism the results were consistently supporting the initial hypothesis with IL-1β rising when accounting in the model for the preceding absence or presence of the *in vivo* first LPS hit (interaction terms “NB” * “hits” (p = 0.048).

### RNAseq approach

#### Whole transcriptome analysis

We reported the genomic landscape of primary fetal sheep microglia in response to LPS using similar quality control methods to confirm the cell culture purity^23^. Here we sequenced at high throughput the whole transcriptome of microglia exposed to LPS and pre-incubated with α7nAChR agonist or antagonist (Fig. 1A). We performed a direct differential analysis of NA versus NB which eliminated the background noise of NC. This approach allowed us to observe the immediate effect of LPS on microglial transcriptome when it is modulated by α7nAChR antagonist versus agonist treatments. We performed 6 differential analyses of microglia exposed to agonist and antagonist (NA, NB, respectively) versus control (NC) and second hit microglia exposed to antagonistic treatment (SHB). In Table 1, we summarized the number of differentially expressed (DE) genes (DEG) found for each differential analysis. Overall, the microglial transcriptome exposed to agonistic and antagonistic drugs revealed a greater amount of DEG than LPS-exposed microglia (latter results were published^23^).

**Table 1:** Differential analysis summary in naïve and second hit microglia after modulation of α7nAChR signaling. Differential analysis of count data was done with the DESeq. 2 package. Differentially expressed genes were selected for padj < 0.1. Up regulation and down regulation represent positive and negative Log2 fold changes, respectively.

	Single hit LPS + B	Second hit LPS + B	
	vs. Single hit Control	vs. Single hit Control	vs. Single hit LPS + B
DE genes*	**2,400**	**7,314**	**7,340**
DE* up regulated	1,432	4,351	4,086
DE* down regulated	968	2,963	3,254
*padj < 0.1			
		**Single hit LPS + A**	
	**vs. Single hit Control**	**vs. Single hit LPS**	**vs. Single hit LPS + B**
DE genes*	**2,007**	**2**	162
DE* up regulated	1,103	2	24
DE* down regulated	904	0	138
*padj < 0.1			

#### Unique transcriptome signature of agonistic and antagonistic stimulation in microglia

**Figure 2:**
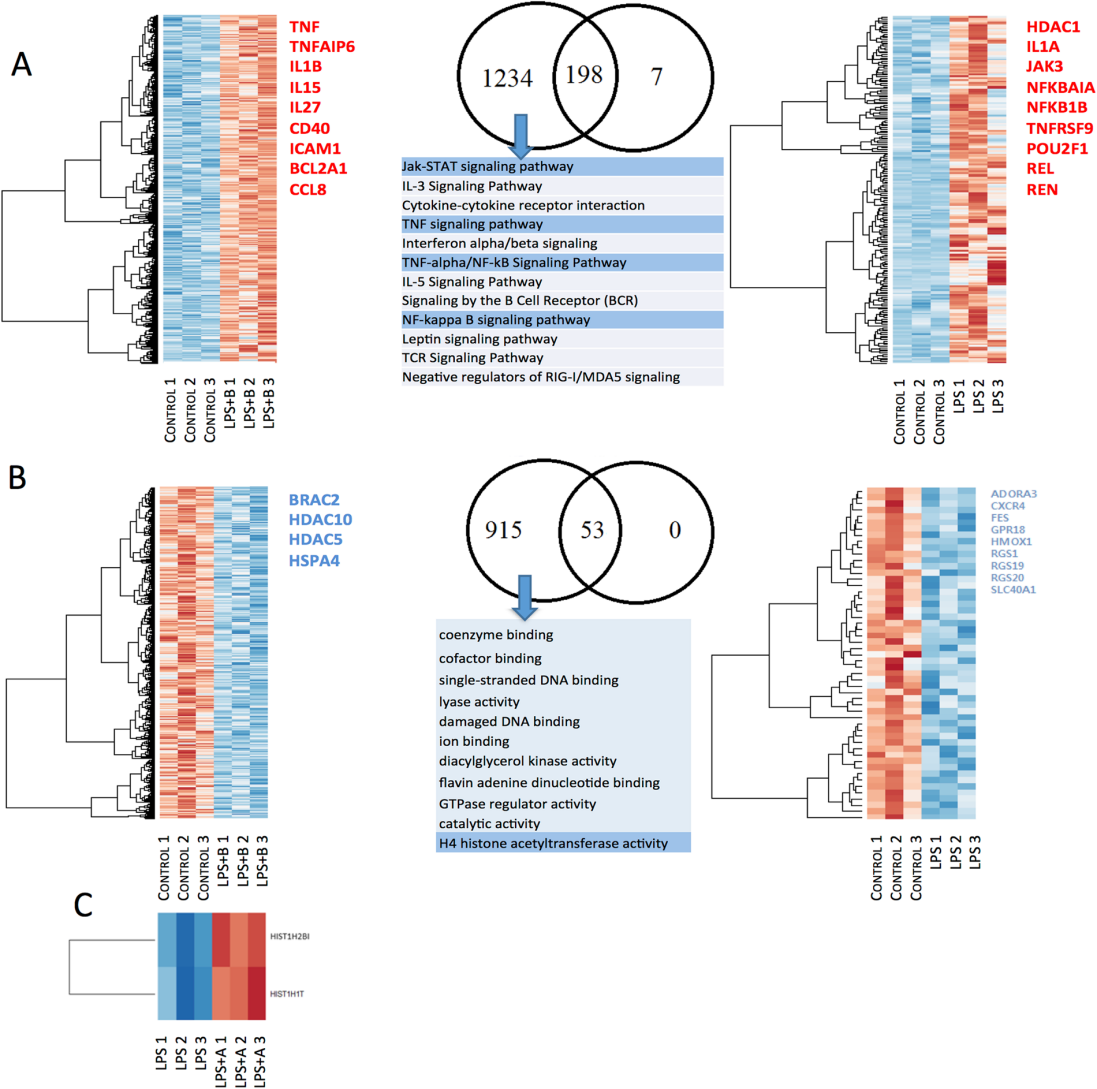
Stimulation of α7nAChR enhances the anti-inflammatory properties of fetal microglia. Differential analysis of the transcriptome of microglia pre-treated with the α7nAChR antagonist α-Bungarotoxin (NB) and the α7nAChR agonist AR-R17779 (NA) compared to controls (NC) and compared to the *in vitro* LPS-treated microglia whose α7nAChR activity was not modulated (NL). The Venn diagrams represent the number of unique genes for each group, and in the middle, the number of common genes. Selected genes of interest are written on the right side of each heat map. (**A**) Microglia treated with the α7nAChR antagonist α-Bungarotoxin recruited more genes involved in the inflammatory pathway. (**B**) α-Bungarotoxin also increased the response in DE down regulated genes. (**C**) NL and NA microglia revealed two DE up regulated histone genes.

We identified 1,432 DEG (padj < 0.1) up regulated genes in NB microglia compared to NL. We compared the population of DE up regulated genes in NB with those in NL microglia: 1,234 genes were unique to NB. The analysis of pathways with Toppcluster revealed that unique genes to NB are members of the Jak-STAT, TNF-α and NFKB signaling pathways (Table 1, Fig. 2A).

968 DE down regulated genes were identified in NB microglia compared to NL. All 53 genes previously identified in NL were also DE down regulated in NB. Thus, NB showed a unique signature of 915 genes. Gene ontology of genes unique to NB revealed that these genes are mostly part of coenzyme binding (GO:0050662 and P = 8.37 × 10^−6^), GTPase regulator activity (GO:0030695 and P = 3.11 × 10^−5^), damaged DNA binding activity (GO:0003684 and P = 2.59 × 10^−4^) and H4 histone acetyltransferase activity (GO:0010485 and P = 9.42 × 10^−4^).

We extracted genes that were uniquely differentially expressed in NB and not found in LPS-exposed naïve microglia (NL vs. NC). We reported up regulation of genes involved in the NFKB and JAK-STAT pathway^23^. A closer look at all genes involved in these two pathways confirmed up regulation of TNF and IL1B. However, in our previous results, the latter two were not differentially expressed under LPS exposure alone. Our current differential analysis of microglia treated with α-Bungarotoxin prior to LPS exposure showed that both TNF and IL1B are differentially expressed and up regulated (padj = 1.17 × 10^−25^ and padj = 8.02 × 10^−88^, respectively), further confirming our hypothesis that antagonistic stimulation of α7nAChR potentiates LPS-triggered microglial inflammation (Fig. 2A). Confirming our previous findings and the notion of the pro-inflammatory effect of blocking signaling through α7nAChR, we found HMOX1 to be progressively stronger down regulated and FBP up regulated due to a second-hit LPS exposure and subsequent pre-treatment with the α7nAChR antagonist (SHB) (Table 2).

**Table 2:** Genetic expression of HMOX1 and FBP after exposure to LPS and signaling through α7nAChR. HMOX1 is progressively stronger down regulated and FBP up regulated due to a second-hit LPS exposure preceded by a pre-treatment with the α7nAChR antagonist α-Bungarotoxin (SHB).

Differential analysis	HMOX1		FBP	
	Log_2_	padj	Log_2_	padj
Second hit LPS vs. naïve LPS (SHL-NL)	**−4.30**	**8.13E-02**	**4.06**	**9.40E-02**
Second hit LPS + B vs. naïve LPS + B (SHB-NB)	**−4.78**	**3.02E-13**	**3.26**	**3.23E-05**
Second hit LPS + B vs. naïve control (SHB-NC)	**−7.07**	**3.92E-40**	**3.06**	**6.86E-04**
Naïve LPS + B vs. naïve LPS + A (NB-NA)	0.17	1.00E + 00	−0.72	8.45E-01

In a similar way, from both differential analyses to baseline NC, we extracted DE down regulated genes unique to NB and not found in NL. Here we found that HDAC10 and HDAC5 are uniquely DE down regulated in NB (padj = 8.81 × 10^−2^, padj = 5.24 × 10^−6^, respectively). Further analysis of HDAC genes is described below (Fig. 2B).

When comparing NL to NA, we did not identify any DE down regulated genes. However, differential analysis of agonist-stimulated microglia rendered a unique signature compared to LPS-treated microglia. We found that two genes were DE up regulated in NA versus NL, ENSOARG00000020076 and HIST1H1T (padj = 1.01 × 10^−10^, padj = 4.98 × 10^−2^, respectively). Per ensemble database, the gene ENSOARG00000020076 corresponds to the HIST1H2BI, a family member of the Histone cluster 1 H2B (Table 3, Fig. 2C).

**Table 3:** Impact of α7nAChR signaling on memory of inflammation. Differential analysis of HDACs and HATs genes, differentially expressed genes (DEGs) are indicated with a bold font (padj < 0.1). In our previous report, we highlighted the potential role of HDAC1, 2 and 6 in memory of inflammation in fetal microglia.

Relevance	Gene	Single hit Control vs.				Single hit LPS vs.	Single hit LPS + B vs.	
		Single hit LPS	Second hit Control	Single hit LPS + B	Single hit LPS + A	Second hit LPS	Second hit LPS + B	Single hit LPS + A
HDAC genes: Potential epigenetic regulators	**HDAC1**	**2.271**	0.145	**3.006**	2.470	0.676	−0.690	−0.533
	HDAC10	−0.242	−0.84	−**1.109**	−0.914	0.116	1.140	0.193
	HDAC11	−0.214	0.867	−1.201	−1.166	0.812	2.368	0.040
	**HDAC2**	−0.299	−**2.746**	0.206	0.256	−2.423	−**2.719**	0.051
	HDAC3	0.045	−0.692	0.211	0.057	0.321	−0.331	−0.160
	HDAC4	1.292	1.502	0.761	0.243	−0.691	0.718	−0.515
	HDAC5	−0.869	0.333	−**1.614**	−1.355	0.501	**0.889**	0.260
	**HDAC6**	−**0.688**	−0.126	−**0.824**	−0.695	−0.43	0.250	0.129
	HDAC7	−0.109	0.643	−0.156	−0.690	−0.486	−0.474	−0.534
	HDAC8	0.336	−0.889	**0.802**	0.653	−2.51	−**2.623**	−0.150
	HDAC9	0.732	1.556	−0.251	−0.565	−1.816	0.296	−0.311
Histone Acetyltransferase 1	HAT1	−**0.379**	−1.639	−0.137	0.431	−0.865	−**1.470**	0.564

#### Effect of α7nAChR agonist and antagonistic drugs on microglial transcriptome

**Figure 3:**
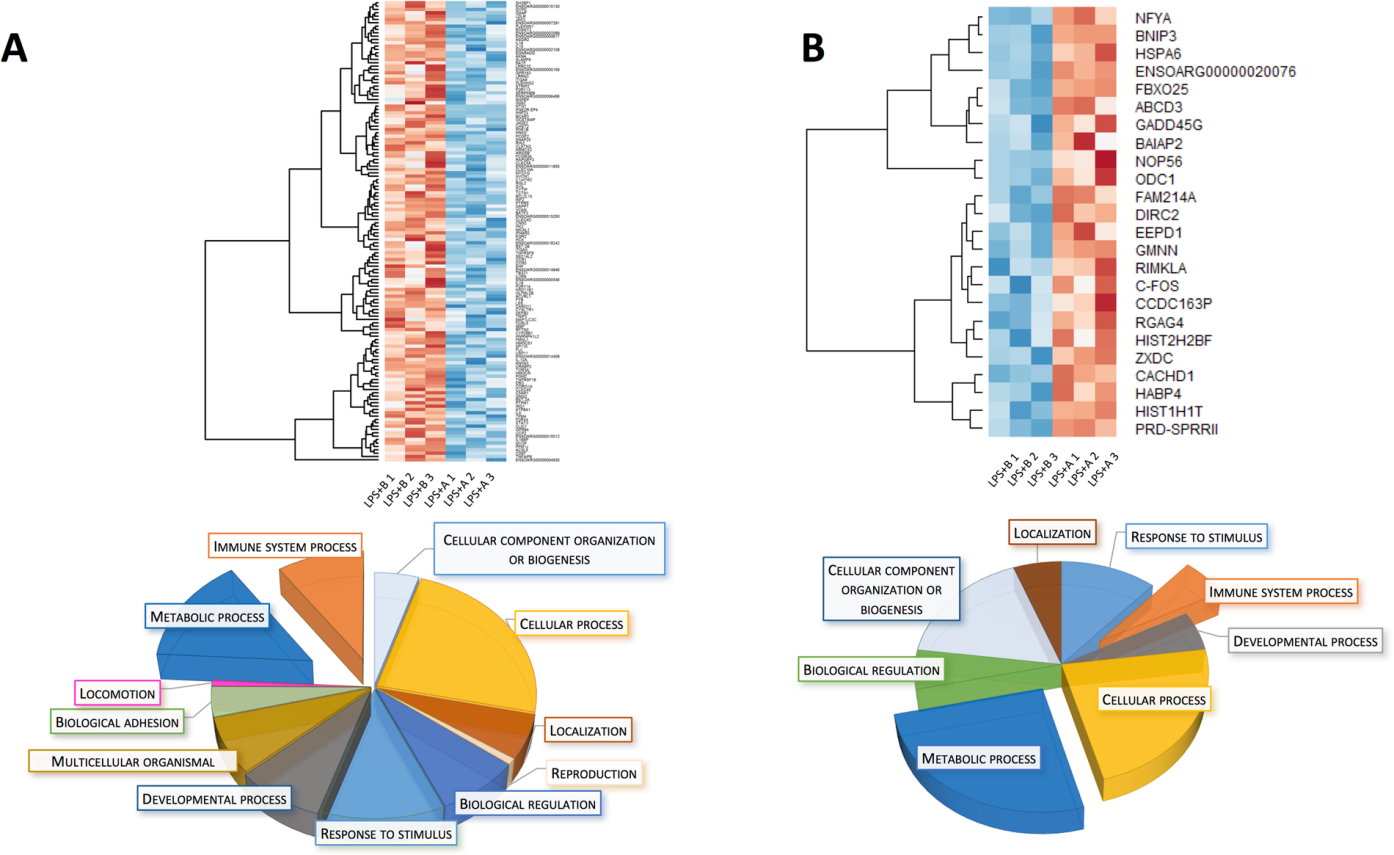
Differentially expressed genes (DEG, padj < 0.1) in agonist (NA) compared to antagonist-pre-incubated LPS-exposed naïve microglia (NB). (**A**) Heat map representation of differentially expressed down regulated genes and gene ontology pie chart. (**B**) Heat map of up regulated genes in NA, compared to NB. Gene Ontology of each set of DEGs is presented as a pie chart at the bottom of each corresponding heat map. Note that “Immune system” and “Metabolic process” are both strongly down and up regulated, possibly referring to different functions being turned off while others are turned on under the influence of cholinergic signaling through α7nAChR.

Our main analysis focused on differences between NB and NA treatment. Our differential analysis of NA versus NB revealed 162 DEG, among which 24 were upregulated and 138 were down regulated (Table 1). Gene ontology of DEG down regulated in NA versus NB showed that DE down regulated genes were associated with the immune system (GO:0002376), however two DE up regulated genes also clustered for the GO terms immune system, HSPA6 and GADD45G (Fig. 3B). In the human genome, the gene HSPA6 codes for the Heat shock 70 kDa protein 6, and GADD45G codes for Growth arrest and DNA damage-inducible protein GADD45 gamma.

**Figure 4:**
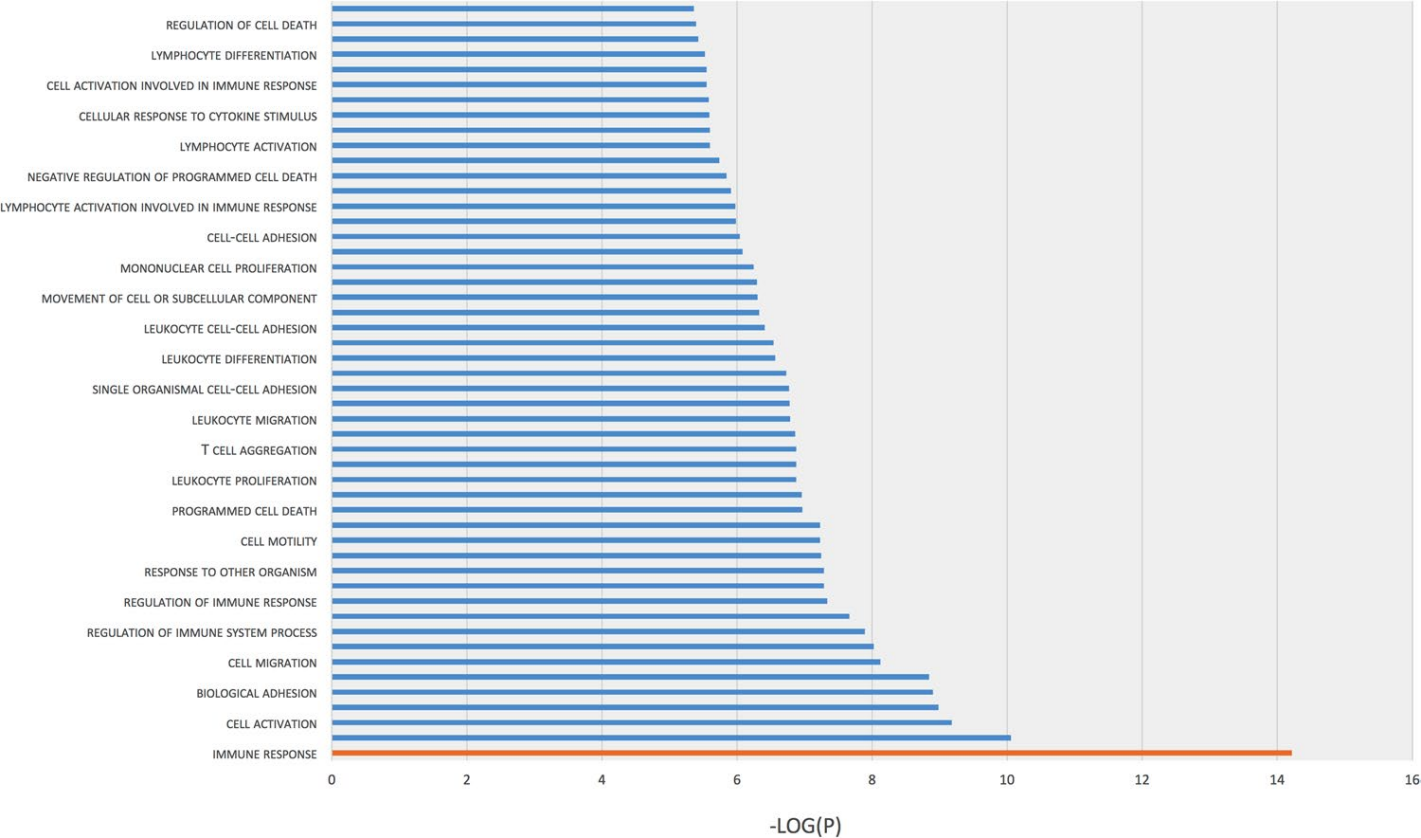
Gene ontology analysis with ToppGene of selected DEGs in agonist (NA) compared to antagonist-preincubated LPS-exposed naïve microglia (NB). Bar graph of 138 down regulated DEGs. Each selected GO term (P < 10^−3^) is represented on a –Log (P) scale.

Interestingly, we noticed that GO terms such as Locomotion (GO:0040011) and Reproduction (GO:0000003) were associated with DE down regulated genes (Fig. 3A). We performed a second GO analysis with ToppGene of DE down regulated genes, selected significant GO terms clusters (P < 10^−3^) and represented these in a bar chart with -Log(P) (Fig. 4). Among DE down regulated genes, the immune response was strongly significant (P = 6.10 × 10^−15^); we also noticed leukocyte migration and inflammatory response among the GO terms clustering (Fig. 4).

Lending support to our hypothesis, these results confirmed the anti-inflammatory effect of the α7nAChR agonistic stimulation on microglia.

#### Modulation of the memory of inflammation by α7nAChR signaling: interference with iron homeostasis

**Figure 5:**
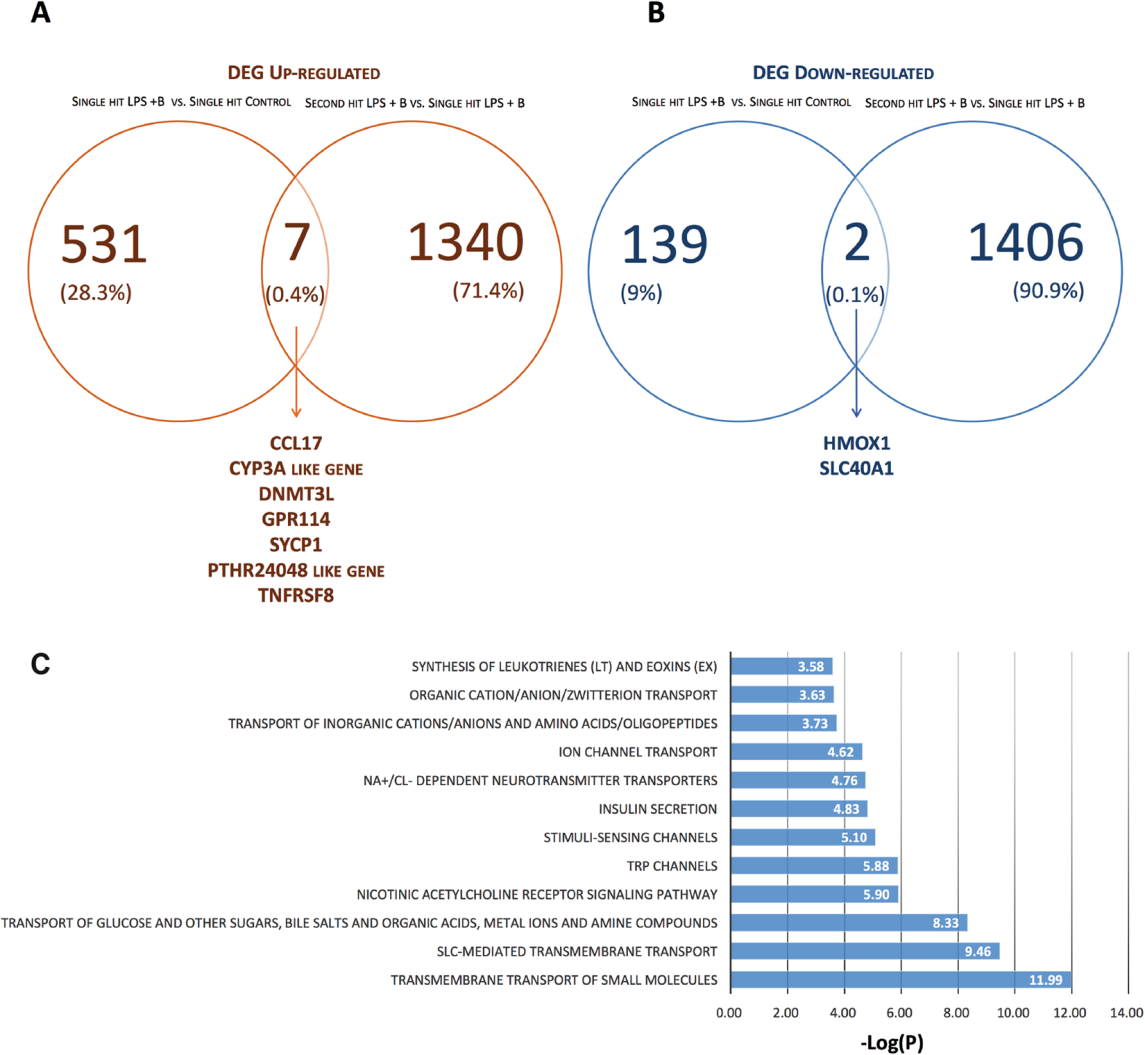
Co-stimulation with α-Bungarotoxin (**B**) shows a unique transcriptome profile in microglia in response to LPS highlighting the contribution of iron homeostasis genes. Uniquely UP (**A**) and DOWN (**B**) regulated differentially expressed genes in second hit α-Bungarotoxin-treated microglia (SHB) are shown for padj < 0.01, Log_2_ > |2|. Each set of the common DEGs is written at the bottom of the Venn diagram. (**C**) Pathways revealed by Gene Ontology of uniquely differentially expressed down regulated genes in SHB; pathways are represented with their -Log(P).

From the two differential analyses, NB versus NC and SHB versus NB, we selected up and down regulated genes with Log_2_ > |2| and represented common genes into a Venn diagram (Fig. 5A,B). A total of 7 genes were DE and up regulated in both analyses, and two genes were down regulated. Interestingly, common down regulated genes were HMOX1 and SLC40A1.

We reported the potential role of heme-oxigenase 1 (HMOX1) during neuroinflammation and will focus here on the gene SLC40A1 (ferroportin)^23^. Using a double-hit model of LPS exposure, we showed that microglia gained memory of inflammation when pre-exposed to LPS *in vivo*, and we also pin-pointed the role of iron metabolism in this process. Here, gene ontology of uniquely down regulated genes in SHB versus NB contained, among pathways affected, metal ions compound (P = 4.64 × 10^−9^) and SLC-mediated transmembrane transport (P = 3.49 × 10^−10^). Similar to our earlier results with HMOX1^23^, this finding now highlights the putative role of solute carrier family (SLC) genes, another key component of the iron homeostasis in neuroinflammation, when cholinergic signaling is perturbed (Fig. 5C).

**Figure 6:**
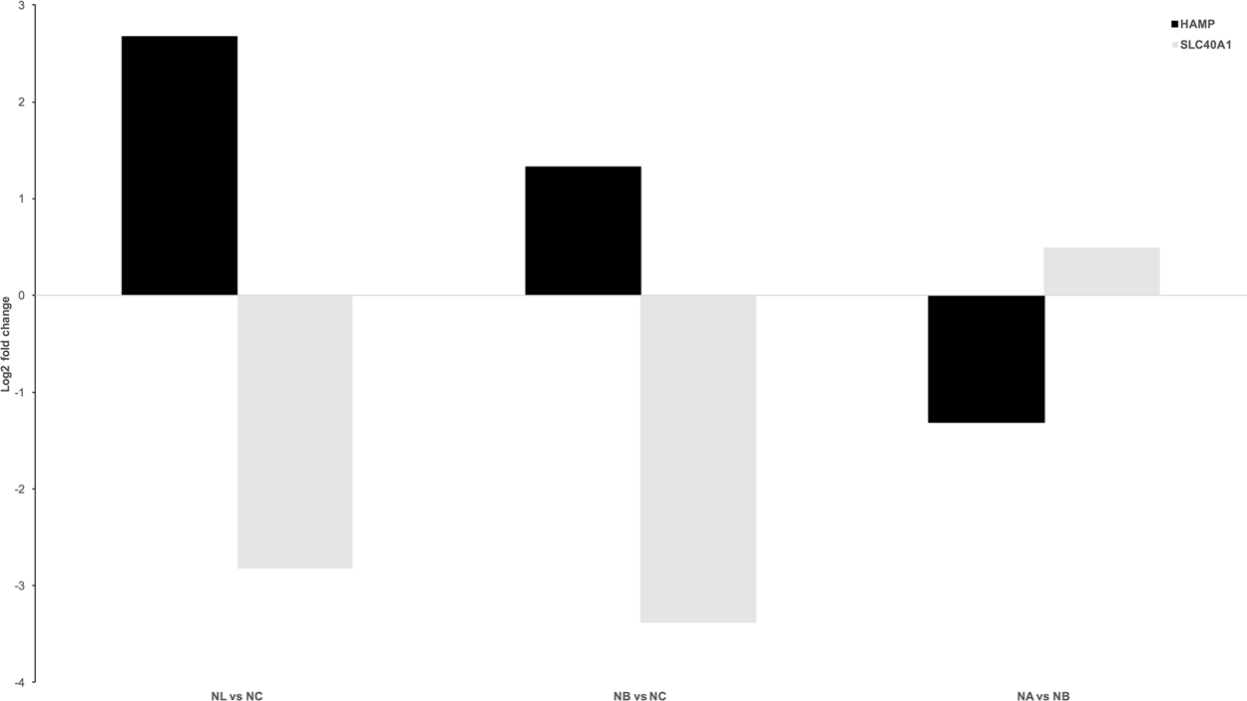
Differential analysis of SLC40A1 and HAMP in naïve, α7nAChR agonist and antagonist-treated microglia. Each differential analysis is noted on the x-axis. The gene SLC40A1 is coding for the protein Ferroportin-1, a transmembrane protein, transporter of iron molecules out of cell.

Hepcidin (HAMP) plays a key role in linking inflammation and iron homeostasis^24^, therefore we examined the RNA transcript level changes of hepcidin and ferroportin in our α7nAChR signaling model (Fig. 6). Differential analyses of NL and NB to baseline and NA vs. NB revealed an opposite pattern of expression of HAMP and SLC40A1 in agonist-treated microglia, wherein ferroportin was up regulated (Log_2_ = 0.49) and hepcidin was down regulated (Log_2_ = −1.31). However, only ferroportin was differentially expressed in NL and NB versus NC (padj = 9.79 × 10^−4^, padj = 4.26 × 10^−5^, respectively). It was not found to be differentially expressed in NA versus NB. Despite the strong Log_2_ fold changes of RNA transcript, HAMP was not differentially expressed in neither of these three comparisons.

Similar to our findings in naïve microglia^23^ and lending support to the epigenetic mechanisms of such neuroinflammation memory, here we also found HDAC1 up regulation following LPS exposure to be potentiated by pre-treatment with α7nAChR antagonist (NC-NB). This supports the notion that blocking α7nAChR signaling has pro-inflammatory effects not only on the level of cytokine secretion, but also on the level of strengthening the inflammation memory. HDAC6 behaved in the opposite direction of HDAC1, again similar to what we reported for LPS alone and potentiated by the α7nAChR antagonism (Table 3).

#### Validation by quantitative RT-PCR

**Figure 7:**
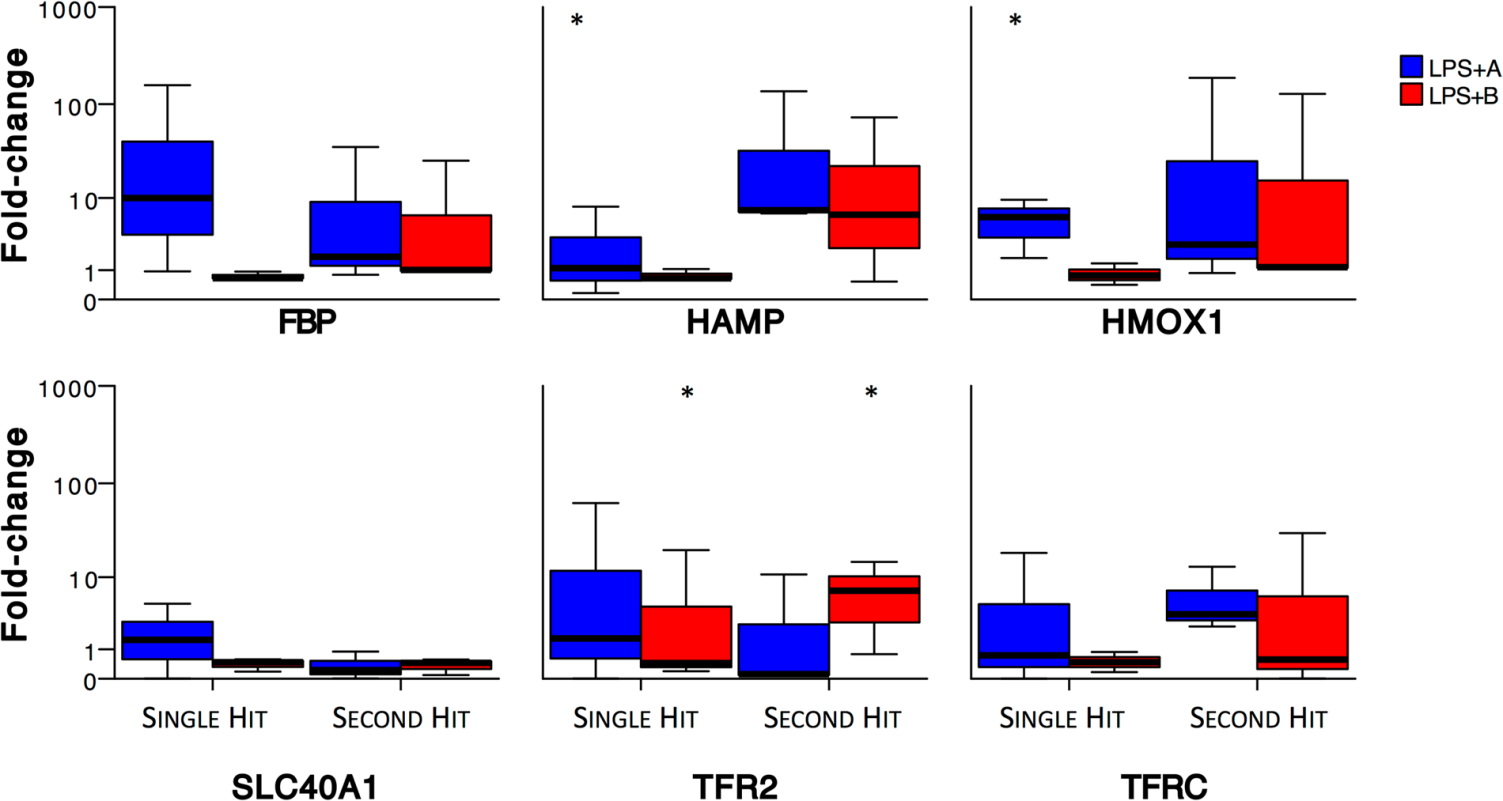
Validation of RNAseq findings by qRT-PCR. *HAMP, SLC40A1, TFR2, TFRC, HMOX1* and *FBP* are shown normalized to the individual levels of LPS exposure alone. Y axis is logarithmic. *, p < 0.05 using GEE modeling. N = 3 in each group. Note the pronounced effects of the LPS second hit on the transcript patterns regardless of the cholinergic manipulation (p < 0.023) as well as the differential effects of LPS hits under cholinergic agonism (LPS + A) or antagonism (LPS + B) on the key iron transcripts HAMP, HMOX1 and TFR2 (details in text).

We validated the key targets of iron and energy homeostasis, *HAMP, SLC40A1, TFR2, TFRC and HMOX1*, by qRT-PCR using GEE model with main term “transcripts” (Fig. 7). The model effects showed no significance for the main term “hits” (*i.e*., single or second hit, p = 0.503), but were significant for the main terms “groups” (LPS + A or LPS + B) (p < 0.001) and “transcripts” (p = 0.036). We confirmed a significant interaction for “transcripts” * “hits” (p = 0.023), but not for “transcripts” * “group” (p = 0.056). Lastly, there was a significant interaction between all three main terms (“transcripts” *“hits” * “group”, p = 0.04). That suggests a significant effect of cholinergic manipulation during microglial exposure to inflammation on the observed patterns of transcripts representing iron and energy homeostasis and a significant contribution of repeated LPS exposure to these effects. Individually, we found significant parameter estimates for the interaction of “Single Hit” * “LPS + B” * TFR2 (p = 0.009), “Second Hit” * “LPS + B” * TFR2 (p = 0.013), “Single Hit” * “LPS + A” * HAMP (p = 0.015) and “Single Hit” * “LPS + A” * HMOX1 (p = 0.015).

## Discussion

In a unique double hit model of fetal neuroinflammation in a large mammalian brain, we studied transcriptomic changes following modulation of cholinergic signaling through α7nAChR with selective antagonistic drug α-Bungarotoxin and agonistic drug AR-R17779. We previously reported the activation of major inflammatory pathways JAK-STAT and NFKB in naïve microglia exposed to LPS as an inflammatory stimulus^23^. In our current analysis, we demonstrated the enhanced activation of these inflammatory pathways in microglia exposed to LPS and pre-treated with α-Bungarotoxin. Both, transcriptomic activation and protein level IL-1β secretion patterns were enhanced in α-Bungarotoxin pre-treated microglia compared to microglia exposed to LPS alone. Our findings extend the *in vitro* observations in mature rodent primary microglia cultures to a large mammalian developing brain exposed to LPS *in vivo* and *in vitro* ^15^.

We aimed to understand the biological processes in microglia when exposed to AR-R17779. The key finding from the differential analysis of NA versus NB was that genes of the immune response were DE down regulated in NA and most of these genes were up regulated in LPS-treated microglia. Notably, JAK3 was DE down regulated in NA, and C-FOS was up regulated. DE genes up regulated in NA and clustering in the immune system process GO term comprised only HSPA6 and GADD45G. The latter is known to regulate cytokine expression during LPS-induced inflammation^25^, whereas HSPA6 is only induced under severe oxidative stress^26^. Lending support to our hypothesis, our findings in NA versus NB comparisons confirmed the anti-inflammatory effect of the α7nAChR agonistic stimulation in microglia.

The current findings represent the first direct *in vitro* validation of our earlier indirect *in vivo* observations in the fetal sheep brain of the same gestational age when we proposed the existence of a fetal cerebral cholinergic anti-inflammatory pathway with *in situ* evidence of the expression of α7nAChR on microglia^27^.

### Effect of α7nAChR signaling on the FBP/HMOX1 microglial phenotype

We reported HMOX1_down_/FBP^up^ transcriptome phenotype in SHL microglia^23^. We expected that the agonistic stimulation of α7nAChR would attenuate this phenotype. HMOX1 and FBP were both differentially expressed in SHL microglia and this phenotype was sustained with antagonistic treatment. Differential expression in naïve microglia NA versus NB did reveal an opposite expression, wherein HMOX1 was up regulated and FBP was down regulated in NA, however a statistically significant differential expression was not observed. Thus, further studies are needed to validate these results in the SHL microglia stimulated agonistically on the α7nAChR (cf. Methodological considerations).

#### α7nAChR signaling modulates the epigenetic memory of inflammation in fetal microglia

Our findings suggest a potential role of histones in the memory of neuroinflammation^23^. Here, we asked whether epigenetic mechanisms are involved in enhancement and reduction of neuroinflammation in microglia via α7nAChR signaling. Differential analysis of NA versus NL revealed two DE up regulated genes, HIST1H2BI and HIST1H1T, both corresponding to the histone cluster 1 H, strengthening our hypothesis of memory of neuroinflammation sustained by epigenetic factors^23^ and extending it to involve α7nAChR signaling.

Our previous report showed that HDAC1, HDAC2 and HDAC6 were potentially involved in the memory of neuroinflammation. In this study, among selected HDAC genes DE in NB compared to NC, all showed an opposite expression pattern in NA compared to NB. The gene HAT1 was also DE down regulated in our previous report and was up regulated in the agonistically-treated microglia. Of note, similar to our published findings in naïve microglia, here we also found HDAC1 up regulation following LPS exposure and this effect was further potentiated by pre-treatment with α7nAChR antagonist (NC-NB) supporting the notion that blocking α7nAChR signaling has pro-inflammatory effects not only on the level of cytokine secretion, but also on the epigenetic level by strengthening the inflammation memory.

The present findings are supported by studies reporting the distinct consequences observed in mice following psychological stress, which was impacted by HDAC4^28, 29^. Evidence of epigenetic memory can be further explored by testing whether the observed epigenetic regulation enables reversal of the two-hit state. This can be done by using selective histone deacetylase inhibitors. Neuroinflammation has been shown to be regulated by several intricate microRNA-HDAC interactions^30, 31^. Future work will explore the inflammation-regulatory level of microRNAs in the context of the current observations. This may be validated by qPCR.

#### Microglial iron homeostasis may be modulated by α7nAChR signaling: implications for neurodevelopment

To further address mechanisms involved in memory of inflammation, we extracted common DE genes between naive and second hit microglia treated with α-Bungarotoxin. Common DE up regulated genes included CCL17, CYP3A and DNMT3L. CCL17 is known to mediate inflammation in macrophages^32^, while CYP3A (cytochrome P450 3 A) plays a major role in drug metabolism. CYP3A function in microglia is not known. Previous reports cited the up regulation of DNMT3L in TLR3- and TLR4 stimulated microglia^33^, concordant with our results in α-Bungarotoxin treated microglia. We identified two DE down regulated genes, SLC40A1 and HMOX1. In conjunction with published results on HMOX1, we aimed to understand the role of SLC40A1, an iron-regulated transporter, known as ferroportin.

Our data indicate a combination of down regulation of metal ion transporter, ferroportin, with HMOX1. Ferroportin acts as a receptor for hepcidin (HAMP)^34^. Hepcidin production is increased during the inflammatory response with increased binding of hepcidin to ferroportin leading to the internalization and degradation of ferroportin. This mechanism consequently suppresses enteral iron absorption and cellular iron release, whereas a decrease in hepcidin promotes iron uptake. Our results in fetal microglia are concordant with these published data in non-brain cells. Indeed, in naïve microglia (NL) compared to baseline (NC), HAMP was up regulated during inflammation and SLC40A1was down regulated. This expression pattern was reversed in α7nAChR agonist-treated (NA) compared to antagonist-treated microglia (NB).

In addition to the RNAseq, we used qRT-PCR to validate the observation that microglial α7nAChR and iron homeostasis signaling pathways may interact with each other under conditions of neuroinflammation.

Overall, our results suggest that in microglia, during neuroinflammation, iron uptake is not only regulated by hepcidin binding to ferroportin, but also by the level of transcription of ferroportin or transferrin receptor 2 under cholinergic control.

Further studies are needed to clarify the role of hepcidin, ferroportin, transferrin receptors and heme-oxigenase during neuroinflammation and in memory of neuroinflammation. Such studies will help elucidate the mechanism underlying memory of neuroinflammation acquired *in utero* and the neurodevelopmental sequelae such as the neurodegenerative diseases we discuss in the following subsection.

Evidence is emerging that iron overload is intricately involved in cognitive dysfunction, with microglia priming or activation playing a key role in this process^35–37^. Excess intracellular iron may result from postoperative inflammation mediated by hepcidin or age-related iron accumulation under conditions of chronic inflammation. While most work in this area has been done in cultures or rat animal models, to our knowledge, this is the first report of inflammation-triggered changes in iron homeostasis in a larger mammalian brain with high resemblance to human physiology and in patterns of response to injury. We believe the current report is also the first observation of the putative link between cholinergic signaling in fetal microglia and the inflammatory milieu. Remarkably, iron homeostasis genes turn out to be key in determining the phenotype of the double-hit microglia. In light of the known role of iron in cognitive function (and dysfunction), our results raise the possibility that early disturbances in microglial iron metabolism may have profound consequences in fetal and postnatal brain development. Our findings also suggest a possible therapeutic venue to modulate intracellular iron load via the α7nAChR as a means to alter microglial phenotype.

#### Impact of α7nAChR signaling manipulation on complement signaling pathway: putative implications for the early programming of susceptibility to Alzheimer’s disease

Cognitive dysfunction may result not only from iron overload, but also from derangements in the neuronal-glial complement pathway interactions. Hyperactive microglia may prevent physiological synaptogenesis predisposing to Alzheimer’s disease (AD) in later life^38, 39^. The mediating pathway involves microglial-neuronal complement signaling suggesting that microglia could be potential early therapeutic targets in AD prevention or treatment and in other neurodegenerative diseases. The complement genes C1Q and CR3 (also known as CD11B) were involved in the microglia-mediated synaptic loss in a mouse model of AD^38^. Less is known about the function of the complement receptor 2 (CR2, also known as CD21) in neuroinflammation, especially in microglia. One study reports CR2^−/−^ mice to be more prone to neuronal injury with higher levels of astrocytosis following nerve root cord injury. This would suggest a neuroprotective role for CR2^40^. Another study reports CR2^−/−^ mice subjected to traumatic brain injury to exhibit less astrocytosis and less microglial activation which would suggest the opposite role for CR2^41^. In mice, CR1 and CR2 are coded on the same gene and expressed as splice variants. In sheep, however, and other higher mammals these complement genes are coded separately. Hence, more studies are needed to gauge the functional role of CR2 in neuroinflammation, microglia in particular, especially with respect to human neurodegenerative diseases such as AD. Consequently, we conducted a secondary analysis of DE genes in SHB compared to NB for CR2, C1Q chain A and B (C1QA, C1AB, respectively) and Complement component 3 A Receptor 1 (C3AR1) as the best equivalent of CR3 in the annotated genome (Table 4). C3AR1 was the only gene showing a clear opposite pattern between α7nAChR agonist- and antagonist-treated microglia. Specifically, we found that agonistic stimulation of the α7nAChR up regulated C3AR1 compared to antagonistic stimulation. We believe these findings deserve further study because pro-cholinergic drugs are used to treat AD symptoms, but the consequences of cholinergic stimulation on microglial signaling are not well understood. Our results suggest that blocking rather than enhancing α7nAChR signaling in microglia may be beneficial to help slow down synaptic degradation.

**Table 4:** α7nAChR signaling modulates the patterns of the complement C1Q−C3AR1 network activity implicated into microglial−neuronal interactions and pruning. Antagonistic stimulation of the microglial α7nAChR, but not the agonistic stimulation, down regulates both C1Q and C3AR1 expressions while up regulating C2. This is important to study further because pro−cholinergic drugs are used to treat symptoms of the Alzheimer’s disease, but it seems that at least in microglial α7nAChR signaling the opposite effect, blocking the cholinergic signaling, may be beneficial to help slow down synaptic degradation.

Description	Gene	Single hit Control vs.		Single hit LPS + B vs.	
		Single hit LPS	Single hit LPS + A	Second hit LPS + B	Single hit LPS + A
Complement C1Q A chain	C1QA	−0.274	−0.081	−**2.379**	−0.227
Complement C1Q B chain	C1QB	−0.093	0.047	−**2.657**	−0.293
Complement component 3a receptor 1	C3AR1	**1.662**	1.301	−**4.719**	−1.571
Complement receptor type 2	CR2	4.549	1.425	**5.488**	2.509

#### Mechanistic model of interactions between iron homeostasis and α7nAChR signaling pathways in microglia

**Figure 8:**
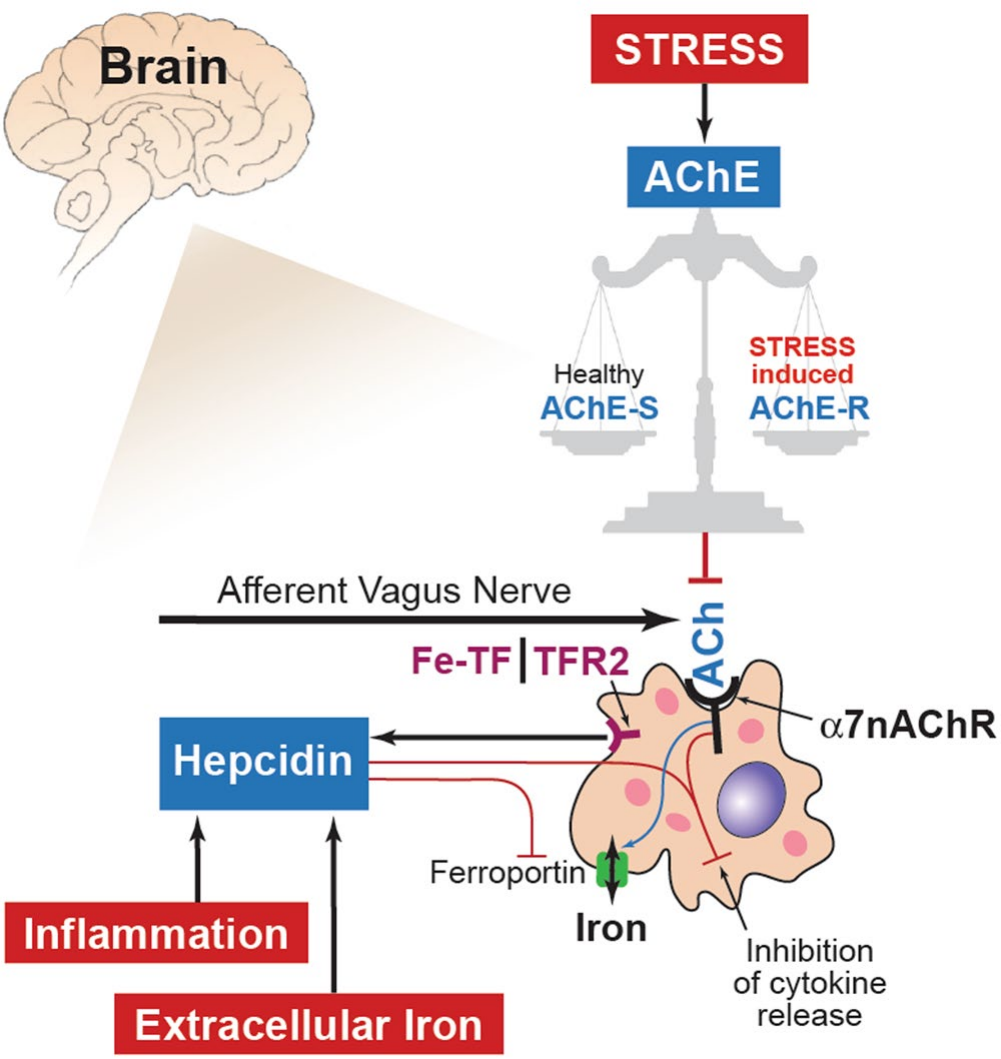
A model of interactions between iron homeostasis and α7nAChR signaling in microglia. Highlighted in red are the three exogenous factors that may be driving the microglial phenotype: inflammation, iron and stress. The former two stimulate hepcidin which in turn acts on ferroportin to be internalized and degraded. This reduces extracellular iron (sensed as Fe-TF, heme transferrin by TFR2, transferrin receptor 2 [shown here simplified as the representative iron sensor receptor] and increases the intracellular iron, a process referred to as iron sequestration. We propose that ferroportin’s membrane localization appears to also be controlled by the α7nAChR signaling (blue arrow). α7nAChR signaling depends on the acetyl choline (ACh) availability. The latter depends on the afferent vagus nerve cholinergic signaling in the brain via a distributed network as well as the non-vagal sources of ACh controlled by ACh esterase (AChE) activity and the availability of dietary choline. Remarkably, a large body of research has shown that AChE activity depends on chronic stress levels, a factor highly relevant in fetal microglia context in particular, because stress is very common in pregnancy. Stress results in shifts of the post-translational modification of AChE from AChE-S splice variant (healthy) to the less stable AChE-R variant.

Based on our findings and the supporting literature, we propose a model of interactions between iron homeostasis and α7nAChR signaling pathways in microglia (Fig. 8). The model highlights in red three exogenous factors that may be driving the microglial phenotype, some more intuitive (inflammation) than others (iron and stress). We briefly outline below the mechanistic connections to the “non-intuitive” factors and refer the interested reader to the cited references for details.

Overall, our findings suggest a yet to be validated signaling pathway between the α7nAChR signaling cascade and the transferrin receptor-hepcidin-ferroportin pathway. Such cascade may counteract, to a degree, the hepcidin’s inhibitory effect on iron release due to the ferroportin internalization and degradation. We propose that this pathway is bidirectional with modulatory effects of hepcidin-ferroportin signaling on α7nAChR signaling. The bidirectional aspect is supported by our surprising observation of the IL-1β secretion profile in the SHA group. *In utero* inflammation may reprogram microglial iron homeostasis toward iron sequestration which in turn diminishes the anti-inflammatory effect of the α7nAChR signaling. At second hit, the resulting net effect on the expression of pro-inflammatory cytokines controlled by NF-kB via the putative α7nAChR - ferroportin – hepcidin signaling network becomes pro-inflammatory which would explain the surprising switch of the IL-1β secretion profile in the SHA group compared to NA group.

The second major factor is the actual availability of the endogenous α7nAChR agonists to drive this pathway. One obvious candidate is the afferent cholinergic anti-inflammatory pathway via the vagus nerve that is comprised to ~80% of afferent fibers and has wide-ranging projections in the brain^27, 42–44^. Another very well researched knob controlling ACh availability in the brain, independent of the vagal cholinergic signaling pathway, is the acetylcholinesterase (AChE). AChE is a key enzyme that regulates the ACh levels^45^. ACh binds to the immune system cells such as microglia in the brain and macrophages in the periphery and decreases their propensity to respond to inflammatory stimuli during healthy (homeostatic) and infectious states (*e.g*., bacterial sepsis)^46, 47^. Under stress, elevated cortisol levels alter AChE gene expression to induce over-production of AChE and replace the major stable AChE splice variant AChE-S by the less stable AChE-R variant^48, 49^. Increased levels of AChE-R have been shown to result in chronic inflammation that ultimately impedes the body’s ability to defend itself against acute infections^50, 51^. AChE-S and –R ratios may influence ACh availability for the microglial α7nAChR signaling. The dependence on stress is particularly intriguing and relevant for the neurodevelopment in the context of fetal microglial physiology, because stress during pregnancy is a very common phenomenon with estimates ranging from conservative 10% to as high as 50% of all pregnant women who report at least one major stress event during pregnancy^52–54^.

#### Methodological considerations

Aside from the lack of biological replicates for SHL microglia, we conducted differential analyses in all other samples. The lack of SHA replicates for sequencing prevented us from studying directly the effect of agonistic drugs on SHL microglia. Thus, further study is needed on the transcriptomic level in SHL microglia stimulated agonistically on α7nAChR.

A limitation of the employed *in vivo – in vitro* experimental system is that it cannot be continuously affected by the surrounding cells and tissues. In particular, the *in vitro* manipulations do not account for the role of the blood brain barrier in the fetal inflammatory response^55^. However, the present double-hit paradigm does show that a memory of inflammation is observed *in vitro* which must be “inherited” from the *in vivo* state of a systemic LPS exposure. This indicates a physiologically relevant *in vivo* mechanism influencing the microglial function.

## Methods

### *In vitro* microglia culture protocol

Fetal sheep brain tissues were obtained during sheep autopsy after completion of the *in vivo* experiment to conduct the *in vitro* study. The non-instrumented, untreated twins were designated “naïve” (no LPS exposure *in vivo*). Instrumented animals that received LPS *in vivo* were used for second hit LPS exposure *in vitro*. Fetal sheep microglia culture protocol was adapted from an established human adult and fetal microglia culture protocol that was modified to include a myelin removal step following the high-speed centrifugation^56^. Briefly, fetal sheep cells were plated on poly-L-lysine (PLL)-coated tissue culture flasks at a concentration of 2 × 10^6^ cells /ml in DMEM with 5% heat-inactivated fetal bovine serum (Gibco, Canada Origin), 1% penicillin/ streptomycin, and 1% glutamine (5% DMEM), in which microglia grow best^56^. Cells were allowed to incubate for seven days at 37 °C, 5% CO_2_, followed by a media change by centrifugation and the addition of re-suspended cells back to the culture flask. Cells were continued to incubate for seven more days with 5% DMEM at 37 °C, 5% CO_2_, before the floating cells were collected. After carefully collecting the floating microglia to avoid contamination with astrocytes and oligodendrocytes, the cells were incubated in 24-well plate at 1 × 10^5^ cells/mL with 5% DMEM for another 4-5 days, and then treated with or without LPS (100ng/ml, Sigma L5024, from E coli O127, B8) for 6 h. Cell conditioned media were collected for cytokine analysis, 0.5 ml TriZol per well added for RNA extraction.

To verify microglia purity, a portion of floating cells was cultured in 24-well plate under the above conditions for flow cytometry analysis (see below). The cell morphology was documented with light microscopy. Another portion of floating cells was plated onto Lab-Tek 8 well chamber glass slide (Thermo Scientific) and treated with or without LPS for immunocytochemistry analysis.

### Cell culture

Microglia isolation and culture were described in detail elsewhere^23^. Briefly, prior to exposure to LPS, cells were pre-treated for 1 hour with either 10 nM AR-R17779 hydrochloride (Tocris Bioscience Cat# 3964), a selective α7nAChR agonist, or 100 nM α-Bungarotoxin (Tocris Bioscience Cat# 2133), a selective α7nAChR antagonist. Optimal dose of AR-R17779 (A) or α-Bungarotoxin (B) was chosen based on a dose-response experiment with LPS; AR-R17779 hydrochloride was dissolved in DMSO into a stock solution. α-Bungarotoxin was reconstituted with culture media into a stock solution and underwent serial dilutions. AR-R17779 and α-Bungarotoxin preparations were added well by well; the same volume of vehicle (either DMSO or cell culture media) was added in control wells. Therefore, in a complete cell culture experiment, we had four experimental groups: Control (naïve control or NC), LPS (naïve LPS or NL), LPS + B (naïve LPS + B or NB) and LPS + A (naïve LPS + A or NA). Second hit cell cultures were designed with the same pattern and divided into four experimental groups: Control (SHC), LPS (SHL), LPS + B (SHB) and LPS + A (SHA).

### Measurements of inflammatory responses

#### Measurement of cytokines in plasma and cell culture media

Cytokine concentrations in cell culture media (IL-1β) were determined by using an ovine-specific sandwich ELISA. Briefly, 96-well plates (Nunc Maxisorp, high capacity microtitre wells) were pre-coated with the capture antibody, the mouse anti sheep monoclonal antibodies (IL-1β, MCA1658, Bio Rad AbD Serotec) at a concentration of 4 µg/ml on an ELISA plate at 4 °C overnight. After 3 times wash with washing buffer (0.05% Tween 20 in PBS, PBST), plates were blocked for 1 h with 1% BSA in PBST for plasma samples or 10% FBS for cell culture media. Recombinant sheep proteins (IL-1 β, Protein Express Cat. no 968-405) were used as ELISA standard. All standards and samples (50 µl per well) were run in duplicates. Rabbit anti-sheep polyclonal antibodies (detection antibody IL-1β, AHP423, Bio Rad AbD Serotec) at a concentration of 4 µg/ml were applied in wells and incubated for 30 min at room temperature. Plates were washed with washing buffer for 5-7 times between each step. Detection was accomplished by assessing the conjugated enzyme activity (goat anti-rabbit IgG-HRP, dilution 1:5000, Jackson ImmunoResearch, Cat. No 111-035-144) via incubation with TMB substrate solution (BD OptEIA TMB substrate Reagent Set, BD Biosciences Cat. No 555214); colour development reaction was stopped with 2 N sulphuric acid. Plates were read on an ELISA plate reader at 450 nm, with 570 nm wavelength correction (EnVision 2104 Multilabel Reader, Perkin Elmer). The sensitivity of IL-1β ELISA for media was 41.3 pg/ml. For all assays, the intra-assay and inter-assay coefficients of variance were < 5% and < 10%, respectively.

#### RNAseq approach

The overall experimental design was divided into three phases: sequencing, quantification and discovery (Fig. 1A). RNA extraction and RNA quantification: Total RNA was extracted from cultured microglia using TRIzol Reagent (Life Technologies). RNA quantity and quality (RNA integrity number, RIN) was established by using a RNA Nano Chip (Agilent RNA 6000 Nano Chips) with Agilent 2100 BioAnalyzer. All samples except SHA had an acceptable RIN value ranging from 6 to 8.5. A total of 12 naïve microglia cultures from four sets of replicates was selected for RNA sequencing at high throughput, as well as three second hit microglia cultures, including SHC, SHL and antagonist-exposed microglia (SHB). Second hit microglia further exposed to agonistic drugs (SHA) were not sequenced due to low RIN. This is left for future studies.

RNAseq libraries were prepared using Illumina TruSeq RNA Sample Preparation v2 kit (Illumina) and quality control was performed on the BioAnalyzer. Single-end 50-bp sequencing was performed at high throughput on an Illumina HiSeq. 2500 at the CHU Ste-Justine Core Facility Sequencing Platform.

### RNAseq data analysis

#### Reads alignment to the reference genome

To maximize the number of genes covered, raw data were mapped to the reference genome of the sheep *Ovis aris* v3.1 from NCBI and Ensembl (GCA_000298735.1) as the transcriptome reference. Index of the reference fasta file was built with Bowtie2^57^. We then trimmed the adaptor of the fastQ files with TrimGalore, and mapped reads to the reference with Tophat2^58^. From the aligned reads from Tophat2, the number of reads per gene was counted with HTseq and assembled into a matrix containing the read count of each gene per sample^59^.

#### Normalization and transcriptome analysis

In order to find differentially expressed genes we used DESeq. 2 to normalize the dataset, generate Log_2_-fold changes and adjacent P values (padj)^60^. We performed 6 differential analyses of microglial transcriptome (Table 1). After stimulation through α7nAChR with agonistic and antagonistic drugs, we aimed to measure changes at the transcriptome level. Thus, we eliminated the background carried by exposure to LPS alone by comparing directly NA read count variation to NB. A gene was considered differentially expressed if its adjacent p-value was strictly lower than 0.1. Pools of up and down regulated genes and differentially expressed genes were clustered and visualized into heat maps, generated in R using the log_2_ normalized counts and the heatmap.2 method of the gplots library^61^.

#### Gene selection and Gene Ontology (GO)

The sheep genome is not yet supported by most gene ontology platforms; therefore, downstream analyses were performed with orthologs in the human genome *Homo sapiens*. To select the relevant genes among the up regulated and down regulated genes, we performed gene enrichment analysis for biological process and molecular function with ToppGenes and FDR < 0.05^62, 63^. Bar diagram of significant GO terms (P < 10^−3^) was presented on a –Log (P) scale. Protein-protein interaction networks were generated with the STRING database and disconnected nodes were not represented^64^. Gene Ontology was also performed in parallel with PantherDB and only biological processes were presented in the pie charts^65^.

#### Validation of RNAseq data by real-time quantitative RT-PCR

The expression profiles of differentially expressed genes related to inflammation and iron metabolism were validated by real-time qRT-PCR. Total RNA (50ng) was subjected to cDNA synthesis using a qScript cDNA SuperMix (Quanta BioSciences) at 25 °C for 5 min, 42 °C for 30 min and 85 °C for 5 min. The mRNAs of the genes *HAMP, SLC40A1, TFR2, TFRC, HMOX1* and *FBP* were quantified by qRT-PCR using the AB SYBR Select MasterMix Kit (Applied Biosystem) with StepOne Plus Real-Time PCR Systems (Applied Biosystems, V2.2.2). PCR was implemented as per the manufacturer’s protocol. The mRNA relative expression was calculated by the 2^−ΔΔCt^ method over housekeeping gene *GAPDH* compared to baseline or LPS-treatment depending on the experimental design^66^. Sheep-specific *HAMP, SLC40A1, TFR2, TFRC and HMOX1* primers were designed with primer3^67^, *FBP* and *GAPDH* primers were designed using Integrated DNA Technologies online tool and primers are listed in Table S1.

### Statistical analyses

GEE modeling approach was used to assess the effects of LPS and drug treatments. For IL-1β, we used a linear scale response model with LPS/drug treatment group (main term “group”) and presence or absence of second hit exposure (main term “hits”) as predicting factors to assess their interactions using maximum likelihood estimate and Type III analysis with Wald Chi-square statistics. For qRT-PCR data, *HAMP, SLC40A1, TFR2, TFRC and HMOX1* expression levels in response to drug treatment followed by LPS exposure were rendered as fold changes of the individual responses to LPS treatment alone and assessed as an additional main term “transcripts” and for interactions with the other two terms in a similar GEE model. SPSS Version 21 was used for these analyses (IBM SPSS Statistics, IBM Corporation, Armonk, NY). Significance was assumed for p < 0.05. Results are provided as means ± SEM or as median {25–75} percentile, as appropriate. Not all measurements were obtained for each animal studied.

### Study approval

This study was carried out in strict accordance with the recommendations in the Guide for the Care and Use of Laboratory Animals of the National Institutes of Health. The respective *in vivo* and *in vitro* protocols were approved by the Committee on the Ethics of Animal Experiments of the Université de Montréal (Permit Number: 10-Rech-1560).

### Data Avaliability

All RNAseq data is available under the GEO accession number GSE101857.

## Electronic supplementary material

Table S1
