# Supplementary material for: α7 nicotinic acetylcholine receptor signaling modulates the inflammatory phenotype of fetal brain microglia: first evidence of interference by iron homeostasis
Source: Sci Rep. 2017 Sep 6;7:10645. doi: 10.1038/s41598-017-09439-z (PMC5587535; doi:10.1038/s41598-017-09439-z)
Supplement: Supplementary file 1 — Table S1 [file 41598_2017_9439_MOESM1_ESM.doc]

**α7 nicotinic acetylcholine receptor signaling modulates the inflammatory phenotype of fetal brain microglia: first evidence of interference by iron homeostasis**

**M. Cortes^2*^, M. Cao^1*^, H.L. Liu^1^, C.S. Moore^3^, L.D. Durosier^1^, P. Burns^4^, G. Fecteau^4^, A. Desrochers^4^, L. B. Barreiro^5^, J.P. Antel^3^, M.G. Frasch^1,2,6^**

*^1^Dept. of Obstetrics and Gynaecology and Dept. of Neurosciences, CHU Ste-Justine Research Centre, Faculty of Medicine,*

*^2^Animal Reproduction Research Centre (CRRA), Faculty of Veterinary Medicine, Université de Montréal, Montréal, QC, Canada;*

*^3^Neuroimmunology Unit, Montréal Neurological Institute, McGill University, Montréal, QC, Canada;*

*^4^Dept. of Clinical Sciences, Faculty of Veterinary Medicine, Université de Montréal, QC, Canada;*

*^5^Dept. of Pediatrics, CHU Ste-Justine Research Centre, Faculty of Medicine, Université de Montréal, Montréal, QC, Canada;*

*^6^Dept. of Obstetrics and Gynecology, University of Washington, Seattle, WA, USA.*

**Running head:** Fetal microglia α7nAChR signaling

**Address of correspondence:**

Martin G. Frasch

Department of Obstetrics and Gynecology

University of Washington

1959 NE Pacific St

Box 356460

Seattle, WA 98195

Phone: +1-206-543-5892

Fax: +1-206-543-3915

Email: [mfrasch@uw.edu](mailto:mfrasch@uw.edu)

* M. Cortes and M. Cao contributed equally to this manuscript

**Table S1. Primers of quantitative real time RT-PCR analysis.**

| Common name | Gene | Forward | Reverse | Genomic coordinates | Exon (#) |
| --- | --- | --- | --- | --- | --- |
| Hepcidin | HAMP | TGGGAAGGAGAGGAAAGGTT | CGTGTCTGTGAAAAGCGAAA | chr14:45097469-45098753 | 3 |
| Ferroportin-1 | SLC40A1 | TGGGGTGGAAAAGTCAAGAG | CCAAGCACCTCCTTTACCAA | chr2:118635488-118658474 | 8 |
| Transferin Receptor | TFR2 | ATAAGCACAGGTGGGTCCAG | GACCTCAGCAGGACAAGAGG | chr24:35822789-35833934 | 10 |
| Transferin Rc. Complement | TFRC | CGGTCATTGATCTCCCTCAT | TCTTGCCCCCTTTTATCAGA | chr1:188913856-188935549 | 2 |
| Heme Oxygenase 1 | HMOX1 | CACCAAGTTCAAGCAGCTGT | CAACCCTGCGAGAAATGTCC | chr3:178683981-178684165 | 3 |
| Fructose-1,6-bisphosphatase | FBP | CGAATGTGACGGGAGATCAA | GGCATGTTTGTCTTCTTCTGAC | chr2:31666200-31666318 | 2 |
| Glyceraldehyde 3-phosphate dehydrogenase | GADPH | TGAGATCAAGAAGGTGGTGAAG | GCATCGAAGGTAGAAGAGTGAG | chr3:207818866-207819075 | 9,10 |
